# Harnessing Plant Microbiomes to Modulate Molecular Signaling and Regulatory Networks in Drought Stress Adaptation

**DOI:** 10.3390/ijms27031139

**Published:** 2026-01-23

**Authors:** Shahjadi-Nur-Us Shams, Md Arifur Rahman Khan, Sayed Shahidul Islam, Afsana Jarin, Md. Nahidul Islam, Touhidur Rahman Anik, Mostafa Abdelrahman, Chien Van Ha, Thayne Montague, Lam-Son Phan Tran

**Affiliations:** 1 Department of Agronomy, Gazipur Agricultural University, Gazipur 1706, Bangladesh; shahjadinur@gmail.com (S.-N.-U.S.); or mdarkhan@ttu.edu (M.A.R.K.); a.jarin9@gmail.com (A.J.); 2 School of Life and Environmental Sciences, Deakin University, Geelong, VIC 3216, Australia; 3 Department of Plant and Soil Science, Texas Tech University, Lubbock, TX 79409, USA; 4 Department of Plant Pathology, Gazipur Agricultural University, Gazipur 1706, Bangladesh; shahidulrased34@gmail.com; 5 Department of Food Engineering, Gazipur Agricultural University, Gazipur 1706, Bangladesh; nahidul.islam@gau.edu.bd; 6 Institute of Genomics for Crop Abiotic Stress Tolerance, Texas Tech University, Lubbock, TX 79409, USA; tanik@ttu.edu (T.R.A.); chien.ha@ttu.edu (C.V.H.); 7 Institute of One Health and Innovation, Lubbock, TX 79415, USA; mosabdel@ttu.edu

**Keywords:** drought tolerance, molecular adaptation, plant–microbe interactions, rhizosphere microorganisms, climate-resilient agriculture

## Abstract

Drought stress is a major abiotic factor limiting global crop productivity by disrupting cellular homeostasis, impairing photosynthesis, and restricting metabolic activity. Plant-associated microorganisms, including rhizobacteria, endophytes, and arbuscular mycorrhizal fungi, play key roles in enhancing drought resilience through molecular, biochemical, and physiological mechanisms. These beneficial microbes modulate phytohormone biosynthesis, enhance osmolyte accumulation, increase organic acid exudation, and activate ROS-scavenging antioxidant pathways. Microbe-mediated regulation of aquaporins, heat shock proteins, and root system architecture further improves water-use efficiency, hydraulic conductance, and stress acclimation. Advances in microbial genomics and systems biology have revealed the molecular drivers of plant–microbe synergism, enabling the development of tailored microbial consortia and next-generation bioinoculants. Complementarily, genetic and genome-guided modulation of drought-responsive regulatory hubs including transcription factors (e.g., DREBs, NACs, MYBs, and bZIPs), signal transducers (e.g., MAPKs and CDPKs), and protective proteins enhances adaptive plasticity under water deficit conditions. This review integrates current molecular insights into drought-induced perturbations in plants and highlights the convergence of microbial interventions and genome-guided strategies in reinforcing drought tolerance. Emphasizing mechanistic frameworks, scalable microbial technologies, and molecular breeding approaches, this work underscores their potential to improve crop resilience in increasingly water-limited environments.

## 1. Introduction

Drought is one of the most significant abiotic stresses limiting crop productivity worldwide, and its impacts are intensifying due to climate change [1]. Rising global temperatures and irregular precipitation patterns have increased the frequency and severity of water-deficit events, posing major threats to agricultural sustainability and food security [1,2,3]. Drought stress negatively affects plant structural, functional, and molecular processes, which subsequently limits crop production. Insufficient water disrupts the cellular dynamics of the plant-water relationship, ultimately affecting overall plant health [4]. Consequently, the arrested plant growth leads to reduced leaf expansion, stomatal closure, and disrupted enzyme activities, as well as decreased carbon dioxide absorption, which collectively contribute to the deterioration of membrane potential. Furthermore, drought-induced disruption promotes the excessive accumulation of reactive oxygen species (ROS) in plant tissues, which triggers lipid peroxidation and membrane disruption while also contributing to the breakdown of essential cellular components [5]. At the molecular level, plant responses to drought involve complex regulatory networks, including transcription factors such as dehydration responsive element binding (DREB) proteins, NAM, ATAF, and CUC (NAC) proteins, myeloblastosis (MYB)-related proteins, and basic leucine zipper (bZIP) proteins, as well as signaling pathways mediated by mitogen-activated protein kinases (MAPKs) and calcium-dependent protein kinases (CDPKs) [3,4,5]. In past years, several strategies have been taken to compensate for the drought-induced yield loss, where most were focused on traditional breeding and genetic engineering approaches [6]. The primary obstacle to implementing these methods is their high cost, labor-intensive procedures, and the extended time required for meaningful results.

The utilization of plant-associated microorganisms has emerged as an increasingly popular, appealing, and economically viable approach for alleviating drought stress. Several studies show that plant microorganisms, including actinomycetes and mycorrhiza with plant growth promoting rhizobacteria (PGPRs), play essential roles in both directly and indirectly supporting plant growth and resistance under drought conditions [7]. Passive mechanisms, which indirectly support plant growth by mitigating pathogen-induced growth inhibition rather than actively producing bioactive compounds, help maintain overall plant development under water-limited conditions [8]. During direct activation, microorganisms improve plant resilience by producing growth-promoting compounds and hormones that enhance nutrient uptake, water movement, and the synthesis of protective osmolytes and antioxidants [9]. By colonizing plant roots and establishing stable interactions, microbes regulate host gene expression, enzyme activities, and water uptake efficiency, enabling plants to better withstand prolonged water deficit [10,11]. Integration of omics approaches, epigenetic regulation, and genome-guided breeding strategies has provided insights into how these pathways coordinate physiological and biochemical adaptations [12,13]. The synergistic application of microbial interventions alongside molecular and genetic approaches holds promise for developing crops with enhanced drought resilience. This review provides a comprehensive overview of the molecular mechanisms underlying plant responses to drought stress and highlights the role of plant-associated microbiomes in modulating these responses. By linking molecular biology, genetics, and microbial ecology, it aims to offer a conceptual framework for leveraging plant–microbe interactions to improve crop performance under water-limited conditions and support sustainable agriculture in the context of climate change.

## 2. Consequences of Drought Stress on Plants

**Figure 1:**
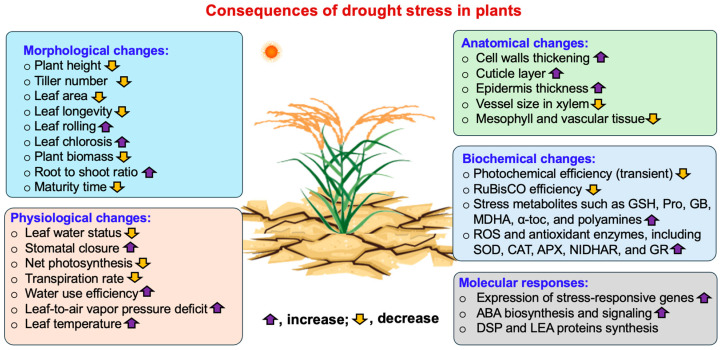
Overview of the consequences of drought stress in plants, including morphophysiological, biochemical, anatomical, and molecular responses. ABA, abscisic acid; APX, ascorbate peroxidase; CAT, catalase; DSP, dehydration stress proteins; GB, glycine betaine; GSH, glutathione; GR, glutathione reductase; LEA, late embryogenesis abundant; MDHA, monodehydroascorbate; NIDHAR, NADPH-dependent enzymes like nitrate reductase; Pro, proline; ROS, reactive oxygen species; SOD, superoxide dismutase; α-toc, α-tocopherol. Figure created using Adobe Photoshop (Adobe Systems, San Jose, CA, USA).

Drought stress significantly restricts plant growth and productivity by modifying morphology, anatomy, physiology, biochemical, and molecular regulation in a systematic manner [14]. Figure 1 provides an overview of the consequences of drought stress in plants, highlighting morphophysiological, biochemical, anatomical, and molecular responses.

During the initial stages of plant development, insufficient soil moisture reduces germination rates, disrupts uniform seedling emergence, and inhibits the elongation of hypocotyls and radicles [15]. Similar constraints persist during subsequent vegetative growth stages. Hossain et al. observed significant decreases in plant height, leaf area, pod and branch number, and overall biomass in drought stress conditions [16]. A concurrent reduction in leaf area index (LAI) further restricts light interception and photosynthetic carbon assimilation, thereby reducing assimilate availability for vegetative and reproductive growth [17]. Disruptions in source–sink relationships also limit resource allocation to developing reproductive tissues, resulting in impaired floral initiation and reduced fertilization efficiency. From a physiological perspective, drought stress markedly reduces photosynthetic efficiency through stomatal closure, decreased mesophyll conductance, and impaired chloroplast function. Reductions in relative water content (RWC) further affect stomatal behavior, transpiration rates, osmotic balance, and carbon fixation capacity, collectively constraining plant productivity under drought stress [18]. At the anatomical level, leaves often exhibit reduced lamina expansion, enhanced deposition of cuticular waxes, and increased leaf rolling, all of which contribute to reduced transpirational water loss [19]. Root systems undergo adaptive remodeling to optimize water acquisition, including deeper rooting, increased root-to-shoot ratios, enhanced suberization, and species-dependent adjustments in xylem vessel diameter, balancing hydraulic efficiency with resistance to cavitation [20]. Biochemically, drought stress promotes excessive accumulation of ROS, such as superoxide radicals (O_2_•^−^), hydrogen peroxide (H_2_O_2_), and hydroxyl radicals (•OH), leading to lipid peroxidation, protein oxidation, and nucleic acid damage. These oxidative processes destabilize cellular membranes and inactivate key metabolic enzymes [21]. To counteract oxidative damage, plants activate antioxidant defense systems, including enzymatic components such as superoxide dismutase (SOD), catalase (CAT), peroxidase (POD), and enzymes of the ascorbate–glutathione (AsA–GSH) cycle, along with non-enzymatic antioxidants [22]. Drought-induced metabolic disruption also involves reduced nitrogen assimilation and impaired carbohydrate partitioning, which further limit energy availability for growth and development [23]. At the molecular level, drought stress perturbs signaling and regulatory networks essential for stress perception and adaptation. Water deficit conditions lead to dysregulated activation of abscisic acid (ABA)-, Calcium ion (Ca^2+^)-, and MAPK-dependent signaling pathways, thereby impairing the coordination of downstream stress responses [18]. Alterations in transcription factor networks, including DREB, NAC, and MYB families, affect the expression of stress-responsive genes encoding aquaporins, dehydrins, and late embryogenesis abundant (LEA) proteins. In addition, drought stress influences epigenetic regulation by modifying DNA methylation patterns and small RNA profiles, which can destabilize chromatin structure and compromise the maintenance of long-term gene expression [24]. These molecular disruptions reduce signaling precision, impair metabolic efficiency, and weaken plant adaptive capacity during prolonged periods of water-limited conditions.

Collectively, drought stress imposes multifaceted constraints on plant growth, spanning morphological, physiological, biochemical, anatomical, and molecular levels. These effects, including reduced germination, impaired photosynthesis, oxidative damage, altered root and leaf architecture, and dysregulated stress-responsive signaling pathways, compromise plant development and productivity. Understanding these consequences provides a foundation for targeted interventions, as plants rely on both intrinsic molecular mechanisms such as ABA, Ca^2+^, and MAPK signaling pathways, transcription factor regulation, and osmoprotectant accumulation. The integration of physiological insights with molecular and microbial strategies, as detailed in Section 3, highlights potential avenues for enhancing drought tolerance and sustaining crop performance under water-limited conditions.

## 3. Genetic and Molecular Signaling Pathways Under Drought Stress

Water scarcity activates a complex array of genetic and molecular signaling pathways that enable plants to adjust to water restriction. Membrane-bound receptors play a crucial role in coordinating ABA signaling pathways, Ca^2+^-dependent signaling, and MAPK cascades, which regulate transcriptional reprogramming in response to initial drought perception [25]. These signaling cascades regulate the expression of stress-responsive genes such as dehydrins, LEA proteins, ROS-scavenging enzymes, and genes related to osmoprotectant biosynthesis, thus protecting cellular integrity and maintaining metabolic balance under water-stressed conditions [26]. The following subsections present a focused analysis of each signaling pathway, emphasizing their molecular interactions and contributions to drought-adaptive phenotypes.

### 3.1. ABA Signaling Pathway

ABA is a critical lipophilic phytohormone that functions as a principal signaling molecule during drought stress. Under control conditions, endogenous ABA levels are maintained at moderate concentrations through a balanced interplay between biosynthesis and catabolism. Drought stress conditions rapidly disrupt this equilibrium by inducing 9-cis-epoxycarotenoid dioxygenase 3 gene *NCED3* and related carotenoid cleavage genes while repressing *ABA 8′-hydroxylase*, leading to a substantial (10–50-fold, species- and tissue-dependent) increase in ABA concentration in xylem sap [27]. Accumulated ABA binds to PYR/PYL receptors, inhibiting PP2C phosphatases and activation of SnRK2 kinases, which triggers rapid transcriptional reprogramming for early drought adaptation. Functional evidence from transgenic studies supports this pathway; for example, expression of *PeCPK10* in Arabidopsis enhances drought tolerance by strengthening ABA-dependent activation of *RD22* and *COR15A* [28].

### 3.2. Calcium Signaling Pathway

Calcium (Ca^2+^) functions as a secondary messenger that transduces drought-induced signals into specific downstream responses. In well-hydrated cells, cytosolic Ca^2+^ concentrations are maintained at approximately 100–200 nM. Drought stress induces transient Ca^2+^ elevations that activate Ca^2+^-responsive components including calcium-dependent protein kinases (CDPKs), calcineurin B-like proteins (CBLs), and CBL–CIPK complexes [22]. These Ca^2+^-mediated modules regulate ion transport, osmolyte synthesis, and stomatal closure, thereby complementing ABA-dependent responses [29].

### 3.3. Protein Kinase Signaling Networks

Protein kinase networks integrate diverse drought-induced signals into coordinated transcriptional and metabolic responses. Key kinase families include CDPKs, MAPKs, and CIPKs, which respond to specific cues and execute pathway-specific roles [28,30,31,32,33]. CDPKs directly transduce Ca^2+^ fluctuations to regulate membrane transport, ROS detoxification, and metabolic homeostasis. The MAPK cascade (MAPKKKs → MAPKKs → MAPKs) primarily coordinates phosphorylation events controlling stomatal behavior and stress-induced metabolism. CIPKs act as integrators, linking Ca^2+^ and ABA signaling, with *GmCIPK2* functioning upstream of ABA-responsive elements in soybean [33]. These kinase networks collectively amplify drought-derived signals while maintaining distinct, pathway-specific contributions, preceding transcription factor activation.

### 3.4. Regulatory Gene Expression

Drought induces widespread transcriptional reprogramming, with transcription factors (TFs) serving as central regulatory hubs that coordinate hormonal and kinase-mediated signaling pathways. Among these, DREB and NAC TF families play prominent roles by activating stress-responsive genes that enhance drought tolerance through modulation of multiple molecular pathways. For example, *PvDREB1F* and *PvDREB5A* demonstrate significant induction under drought stress conditions in common beans [34], while *TaSAP5* in wheat [35] and *ScDREB2B-1* in sugarcane (*Saccharum officinarum*) [36] act as key regulators of drought responses. Sequence variation in *DREB2* alleles has also been suggested as a molecular marker for identifying drought-tolerant genotypes. Furthermore, the DREB family has also been proposed as potential genetic markers for the identification of drought-resistant genotypes [36]. NAC TFs further extend this regulatory network; for example, Stress-responsive NAC 1 (*SNAC1*) enhances drought tolerance in rice by regulating downstream targets such as *OsPP18* and *aPP2C* gene. Transcriptome analyses revealed extensive induction of NAC family members under drought stress, with approximately 40 NAC genes identified in rice and 38 in soybean [37,38]. Together, these TFs connect ABA-, Ca^2+^-, and kinase-mediated signals to structural, metabolic, and antioxidant adaptations which is essential for drought resilience.

### 3.5. Heat Shock Protein Pathway

Heat shock proteins (HSPs) are strongly upregulated during drought stress and contribute to cellular protection by preventing protein aggregation and promoting correct folding of nascent or stress-denatured polypeptides [39]. Heat shock factors (HSFs) act upstream by regulating HSP genes, thereby linking stress perception to protein homeostasis. Through stabilization and refolding of damaged proteins, HSPs mitigate drought-induced cellular injury. In addition, HSPs interact with diverse signaling proteins to enhance stress-responsive gene expression and cellular tolerance [40,41]. Expression profiling revealed that *HmHSF05*, *HmHSF12*, and *HmHSF14* genes were predominantly expressed in root tissues, indicating a potential role in root-specific drought responses. Structural modeling of *HmHSF15* uncovered a distinct three-dimensional configuration, while conserved DNA-binding domains were identified across all HSF proteins. Gene ontology analysis further associated *HmHSF* genes with key abiotic stress-related biological processes, highlighting their involvement in drought stress adaptation in *Hibiscus myrtifolia* [41].

### 3.6. Transgenic Evidence Supporting Drought-Responsive Pathways

Under water-deficit conditions, plants rapidly adjust the expression of stress-responsive genes and proteins to sustain growth and development [12]. Several components, including CaPR-10, vacuolar ATPases, small heat shock proteins (sHSPs), dehydrin-like proteins (Cadhn), 11-pyrroline-5-carboxylate reductase (P5CR), and pyrroline-5-carboxylate dehydrogenase (P5CDH), contribute to drought tolerance by maintaining osmotic balance, and reducing ROS-induced damage [42]. Transgenic approaches provide strong functional evidence for these pathways. For example, the oxidative stress-inducible promoter SWPA2 drives overexpression of the *MnSOD* gene from legumes in rice chloroplasts, enhancing drought resistance. Similarly, co-expression of *TsVP* and *betA* in maize significantly improves drought stress tolerance [43]. Mutants deficient in wax accumulation (*dwa1* and *osgl1-2*) exhibit increased drought sensitivity, whereas overexpression of *δ-OAT* enhances drought tolerance in rice by promoting proline accumulation and facilitating ROS detoxification [44,45,46]. Table 1 summarizes representative transgenic plants with enhanced drought tolerance resulting from the transfer of drought-responsive genes from various donor species.

## 4. Effects of Microbes on Drought Stress Alleviation

Beneficial soil microbes significantly mitigate the detrimental effects of drought stress [61]. Plant-associated microbiota, such as rhizobacteria (e.g., *Pseudomonas* and *Bacillus* spp.) and endophytes (e.g., *Azospirillum* and *Enterobacter*), enhance crop adaptation by promoting root architecture, activating stress-inducible genes, and improving water use efficiency under water-limited conditions [62]. Cyanobacteria (blue-green algae) contribute through nitrogen fixation and phosphate solubilization, and the release of phytohormones and amino acids [11]. For example, the *P. argentinensis* strain SA190 increased drought resilience by priming plant genes via the ABA pathway [63]. Endosymbiotic fungi such as *Aspergillus fumigatus*, *A. terreus*, and *Talaromyces variabilis* have been shown to enhance tomato yield, quality, and drought tolerance [64]. Although plant viruses are conventionally regarded as pathogenic agents, emerging evidence indicates that certain viruses can positively modulate plant responses to drought. In mutualistic symbiotic contexts, viruses may enhance host survival by pre-activating physiological and molecular processes that confer improved tolerance to anticipated environmental stress [65,66]. For instance, several plant viruses, including rice tungro spherical virus (RTSV), tomato yellow leaf curl virus (TYLCV), and tomato chlorosis virus (ToCV), have been reported in specific host–virus–environment contexts to enhance drought tolerance by delaying leaf rolling, improving stomatal conductance, and modulating drought-responsive gene expression [61,65,66]. These beneficial effects are highly conditional and may not occur universally across all plant–virus interactions. In some cases, viruses can also induce the expression of pathogen-related enzymatic genes, such as *CesA*/*Csl*, which contribute both to viral defense under certain conditions [66]. The role of microbial species in mitigating the effects of drought stress is presented in Table 2.

**Figure 2:**
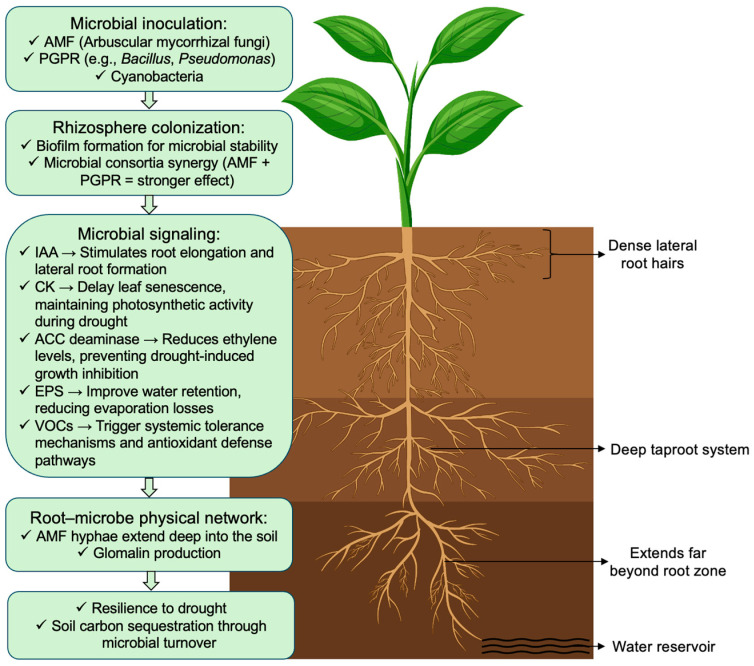
Schematic representation of plant–microbe interactions that enhance drought resilience. Microbial inoculation improves root architecture, water retention, antioxidant defense, and systemic tolerance to drought, while AMF hyphae and microbial consortia extend root reach and enhance soil water acquisition. AMF, arbuscular mycorrhizal fungi; PGPR, plant growth–promoting rhizobacteria; IAA, indole-3-acetic acid; CK, cytokinin; ACC deaminase, 1-aminocyclopropane-1-carboxylate deaminase; EPS, exopolysaccharides; VOCs, volatile organic compounds. Figure created using Adobe Photoshop (Adobe Systems, San Jose, CA, USA).

Understanding microbial interactions under drought stress offers promising avenues for sustainable agriculture. Notably, drought-mitigating microorganisms induce substantial modifications in root system architecture, thereby enhancing soil exploration and improving water uptake under water-limited conditions. Microbial inoculation markedly enhances drought tolerance in plants by improving root morphological and physiological traits (Figure 2). Arbuscular mycorrhizal fungi (AMF), PGPR, serve as effective inoculants by producing a range of phytohormones, primarily auxins, cytokinins, and ethylene, in addition to enzymes such as 1-aminocyclopropane-1-carboxylate (ACC) deaminase and metabolites including exopolysaccharides. These bioactive compounds activate multiple molecular and physiological pathways that collectively contribute to enhanced drought stress tolerance [87]. Following inoculation, rhizospheric microorganisms establish colonies on root surfaces and develop biofilm structures that improve their stability and persistence. Microbial consortia, particularly AMF–PGPR combinations, exhibited synergistic interactions that led to enhanced plant responses relative to the use of individual inoculants alone. Their specific functions as root endophytes include promoting plant growth, enhancing photosynthetic performance, and leaf pigment production. This process leads to an enhanced buildup of compatible solutes, phosphates, and IAA levels, thereby facilitating the preservation of the plant’s overall water balance [88].

Microbial inoculation has been shown to significantly enhance root system development under drought stress in several crop species. For instance, inoculation of pearl millet (*Pennisetum glaucum*) with osmotolerant endophytic bacteria has been shown to enhance root biomass, surface area, and elongation, particularly under severe drought conditions [78]. Similarly, *Azospirillum* inoculation in wheat improved water status by reducing water potential and expanding xylem channels [80]. In sorghum, seed priming combined with *Bacillus cereus* and potassium silicate increased growth performance, RWC, and membrane stability, as indicated by reduced electrolyte leakage under drought stress conditions [89]. Plants respond to drought stress by synthesizing ACC, the immediate precursor of ethylene [90]. Beneficial microorganisms that produce ACC deaminase can sequester plant-derived ACC, which regulates ethylene production and enhances the overall development of the entire plant [91]. In addition, ACC deaminase activity enhanced antioxidant enzyme function, stimulated the production of intracellular osmolytes, and increased leaf pigment content under drought conditions [92]. Several microbial species such as *Pseudomonas aeruginosa*, *Enterobacter cloacae*, *Achromobacter xylosoxidans*, and *Leclercia adecarboxylata* are thought to synthesise ACC deaminase in maize, thereby enhancing the adaptability of the plant under water shortage conditions [93,94,95]. Similar benefits have been observed in grape (*Vitis vinifera*), bell pepper (*Capsicum annuum*), and guar (*Cyamopsis tetragonoloba*) [42].

Beyond ACC-mediated signaling, additional microbial-derived signals contribute to drought tolerance. Volatile organic compounds (VOCs), such as 2,3-butanediol produced by non-pathogenic root-colonizing microorganisms, can induce partial stomatal closure, thereby reducing transpirational water loss. Root colonization by *Bacillus subtilis* and *Pseudomonas chlororaphis* O6 elicits similar responses, with *P. chlororaphis* O6 also exhibiting strong biocontrol activity that further enhances drought tolerance [4]. Moreover, many microorganisms produce hydrophilic biofilms composed of EPS, which improve soil aggregation, enhance root adherence, and increase soil water retention, collectively strengthening plant defense against drought stress conditions [93]. In parallel with biochemical signaling, microbial colonization induces pronounced modifications in root architecture (Figure 2). Microbe-derived IAA stimulates primary root elongation and lateral root formation, resulting in increased root hair density under drought conditions. AMF extend extensive hyphal networks into deeper soil layersthat improves water acquisition beyond the rhizosphere. These interactions enhance hydraulic conductivity and support sustained water uptake during prolonged drought stress [94].

**Figure 3:**
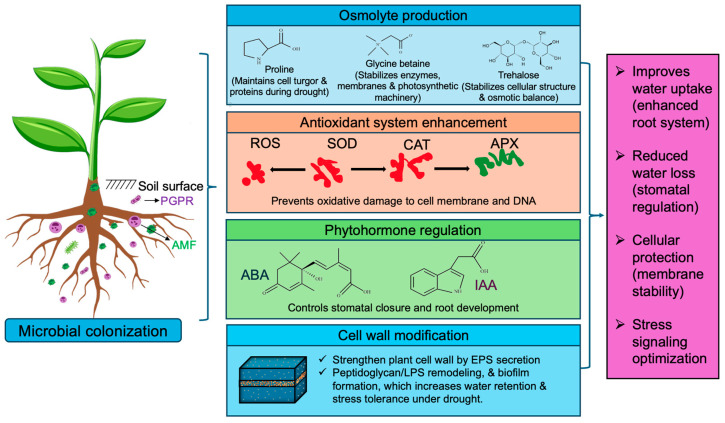
Microbial-mediated enhancement of plant drought tolerance. ABA, abscisic acid; AMF, arbuscular mycorrhizal fungi; APX, ascorbate peroxidase; CAT, catalase; EPS, exopolysaccharides; IAA, indole-3-acetic acid; PGPR, plant growth-promoting rhizobacteria; ROS, reactive oxygen species; SOD, superoxide dismutase. Figure created using Adobe Photoshop (Adobe Systems, San Jose, CA, USA).

Extending the previously defined signaling procedures, microorganisms additionally initiate drought-response pathways (Figure 3). A key microbial involvement is the enhancement of osmolyte production, wherein microorganisms promote the accumulation of compatible solutes, including proline, glycine betaine, and trehalose. These osmolytes contribute to protein stabilization, sustain membrane structural integrity, and maintain cellular turgor pressure under limited water conditions. Root endophytic bacteria, including *Ochrobactrum* sp., *Microbacterium* sp., *Enterobacter* sp., and *E. cloacae*, improved osmotic balance by upregulating proline biosynthesis genes (*SbP5CS1* and *SbP5CS2*), thereby strengthening cellular water retention in sorghum roots [95]. Simultaneously, microbial interactions also strengthen the plant’s antioxidant defense mechanisms by inhibiting enzymes that detoxify ROS. The enzyme combination SOD, CAT, and ascorbate peroxidase (APX) facilitates the detoxification of ROS and safeguards of proteins, nucleic acids, and cell membranes from oxidative damage [96]. In barley, inoculation with *Piriformospora indica* enhanced the expression of genes involved in stress signaling, metabolite transport, and antioxidant defense, thereby reinforcing plant protection against drought-induced oxidative damage [95]. Microbial inoculation increased the activities of SOD and APX, while reducing H_2_O_2_ and malondialdehyde (MDA) accumulation, which are key indicators of oxidative stress and membrane lipid peroxidation under drought stress conditions [90,91].

Regulation of phytohormone homeostasis represents a critical microbial adaptation strategy in arid environments. Beneficial microorganisms modulate the biosynthesis and signaling of gibberellins, cytokinins, ABA, and indole-3-acetic acid (IAA), thereby controlling stomatal behavior, water-use efficiency, and root system architecture under water-limited conditions [97,98]. In particular, ABA-mediated signaling regulates stomatal closure and maintains metabolic stability during drought stress, whereas microbially derived IAA promotes root elongation and lateral root formation. In addition, rhizobacteria produce exopolysaccharides that enhance soil aggregation, improve rhizosphere water retention, and strengthen root–soil adhesion, facilitating sustained water uptake under drought stress [93]. Modifications of microbial cell wall components, including peptidoglycan and lipopolysaccharides, contribute to the stabilization of microbial biofilms in the rhizosphere, ensuring persistent root colonization and indirectly enhancing plant membrane stability and drought tolerance.

## 5. Molecular Interactions in Plant-Microbe Symbiosis for Drought Tolerance

**Figure 4:**
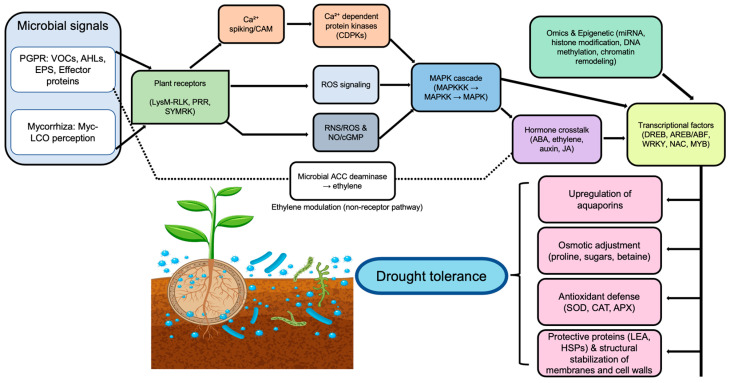
Microbe-induced molecular networks integrating plant stress-responsive pathways under drought. ABA, abscisic acid; ACC, 1-aminocyclopropane-1-carboxylate; AHLs, N-acyl homoserine lactones; APX, ascorbate peroxidase; AREB/ABF, ABA-responsive element-binding protein/ABA-binding factor; Ca^2+^, calcium ion; CAM, calmodulin; CAT, catalase; CDPKs, calcium-dependent protein kinases; DNA, deoxyribonucleic acid; DREB, dehydration-responsive element-binding protein; EPS, exopolysaccharides; HSPs, heat shock proteins; JA, jasmonic acid; LEA, late embryogenesis abundant (proteins); LysM-RLK, LysM receptor-like kinase; MAPK, mitogen-activated protein kinase; MAPKK, MAPK kinase; MAPKKK, MAPK kinase kinase; miRNA, microRNA; Myc-LCO, mycorrhizal lipochitooligosaccharide; MYB, myeloblastosis transcription factor; NAC, NAM, ATAF, and CUC transcription factor family; NO, nitric oxide; PGPR, plant growth–promoting rhizobacteria; PRR, pattern recognition receptor; RNS, reactive nitrogen species; ROS, reactive oxygen species; SOD, superoxide dismutase; SYMRK, symbiosis receptor-like kinase; VOCs, volatile organic compounds; WRKY, WRKY transcription factor family. Figure created using Adobe Photoshop (Adobe Systems, San Jose, CA, USA).

A plant, together with its associated microbial communities, forms an integrated functional entity known as a holobiont. Within this framework, genetic variation exists not only between the host plant and its associated microorganisms but also within the microbial populations themselves. This dynamic and co-evolved relationship influences multiple aspects of plant health, development, and adaptability to environmental fluctuations. The stability and effectiveness of the holobiont depend on coordinated communication systems operating both within microbial communities and between microbes and the host plants [99]. These interactions enhanced disease resistance and stress tolerance, thereby improving the overall resilience of the holobiont. A comprehensive understanding of these complex molecular interactions provides valuable opportunities for developing innovative and sustainable agricultural strategies that exploit beneficial plant–microbe associations to enhance crop productivity. Figure 4 presents a schematic overview of how microbial signaling pathways integrate with plant hormonal and transcriptional networks to promote drought tolerance within the holobiont. The following section highlights key molecular mechanisms underlying these interactions.

### 5.1. Signal Transduction Pathways and Transcriptional Regulation

Signal transduction and transcriptional regulation play central roles in the perception of drought stress signals and the activation of adaptive gene expression in plants. Plant–microbe symbioses, particularly those involving AMF, rhizobia, and PGPR, represent a critical regulatory layer that modulates these molecular pathways under water-deficit conditions. At the molecular level, drought-responsive signaling cascades activate or repress key transcription factor families, including DREB/AP2-ERF, AREB/ABF, WRKY, MYB, and NAC. These transcription factors bind to cis-regulatory elements such as dehydration-responsive elements (DRE/CRT) and ABA–responsive elements (ABRE), thereby controlling the expression of drought-responsive genes [100]. Microbial symbiosis alters these signaling networks, particularly pathways mediated by ABA, Ca^2+^, and MAPKs. Such modifications reshape transcriptional regulatory networks and enable large-scale transcriptional reprogramming, ultimately enhancing plant drought tolerance [12]. For instance, under water-limited conditions, AMF belonging to the phylum *Glomeromycota* significantly improved stomatal conductance in *Poncirus trifoliata* and *Rosmarinus officinalis*. In *Solanum lycopersicum*, AMF inoculation enhanced hydraulic conductivity through modulation of 14-3-3 genes (*TFT1*–*TFT12*) within the ABA signaling pathway, accompanied by changes in phytohormone levels, including strigolactones, jasmonic acid, and ABA [101]. Recent transcriptomic analyses demonstrated that inoculation of wheat with *Funneliformis mosseae* rapidly enhanced aquaporin (AQP) expression in roots during drought stress, while concurrently regulating genes associated with cell wall organization and membrane integrity [102]. Similarly, AMF symbiosis modulated Ca^2+^-dependent signaling pathways, leading to altered expression of CDPKs, MAPKs, CBLs, and CIPKs in maize. These changes collectively influenced cellular signaling, and drought adaptation [103]. Aquaporins constitute a major mechanistic component of AMF-mediated drought tolerance. These pore-forming integral membrane proteins regulate water transport across cellular membranes and are strongly influenced by mycorrhizal colonization [104]. For example, the AQP genes *GintAQPF1* and *GintAQPF2* showed significantly increased expression in the extraradical mycelium of *Rhizophagus irregularis* and in mycorrhizal roots under oxidative stress, indicating direct AMF involvement in water stress regulation [105]. In *Poncirus trifoliata*, *Funneliformis mosseae* consistently upregulated root tonoplast intrinsic protein (TIP) genes (*PtTIP1;2*, *PtTIP1;3*, and *PtTIP4;1*), while downregulating *PtTIP2;1* and *PtTIP5;1*. This differential regulation highlights the functional diversity of AMF in controlling transpiration and root water transport under drought conditions [106]. Compared to AMF, transcriptomic studies addressing PGPR-mediated transcriptional regulation under drought stress remain relatively limited. Nonetheless, available evidence indicated that PGPR inoculation enhanced drought adaptability by suppressing the excessive activation of stress-associated hormonal pathways, particularly those involving ABA and ethylene [107]. Transcriptome profiling of sugarcane roots colonized by *Gluconacetobacter diazotrophicus* revealed that inoculated plants exhibited reduced expression of drought-responsive genes such as *DREB1A/CBF3*, *DREB1B/CBF1*, and *NCED3* homologs, despite active auxin metabolism. While ABA-related pathways were similarly enriched in both inoculated and non-inoculated plants, cytokinin signaling was uniquely upregulated in inoculated plants, indicating hormone-specific modulation by PGPR [108]. Comparable transcriptomic responses were observed in *A. thaliana* inoculated with *Paenibacillus polymyxa* B2. Inoculated plants displayed enhanced transcription of the drought-responsive gene *ERD15* and upregulation of jasmonic acid marker genes (*VSP1* and *PDF1.2*), salicylic acid–regulated gene *PR1*, and ethylene-responsive gene *HEL*, although the magnitude of these responses varied under drought stress conditions [109]. In pepper plants, inoculation with *Bacillus licheniformis* K11 induced the accumulation of six drought-responsive proteins. Notably, the expression of *Cadhn*, *VA*, *sHSP*, and *CaPR-10* genes increased approximately 1.5-fold relative to uninoculated controls [71]. In wheat, inoculation with *B. amyloliquefaciens* 5113 and *Azospirillum brasilense* NO40 enhanced the activity of enzymes involved in the ascorbate–glutathione redox cycle, including APX, thereby priming plants for improved oxidative stress management and reduced drought-induced damage at the whole-plant level [110].

### 5.2. Microbial Regulation of Plant Genes and Hormones Under Drought Stress

Beneficial microorganisms influence plant drought adaptation by modulating drought-responsive gene expression, phytohormone biosynthesis, and associated signaling pathways. This coordinated regulation integrates molecular and hormonal responses, thereby enhancing plant survival under water-limited conditions. Upon drought exposure, plants activate genes involved in water transport, osmoprotectants, and stress signaling to mitigate dehydration-induced damage. Microbial colonization can further amplify these responses by targeting key regulatory nodes within stress-response networks. For instance, *Pseudomonas mandelii* has been shown to enhance nutrient acquisition and facilitate fungal symbiosis in the Mediterranean shrub *Helianthemum almeriense* [111]. Specifically, *P. mandelii* upregulated the plant aquaporin gene TcAQP1, which is essential for the expression of the fungal aquaporin. This interaction likely improves water transport within the plant–fungal continuum, thereby enhancing drought resilience of the symbiotic association. Similarly, Wang et al. demonstrated that inoculation of tomato cultivars with *Bacillus amyloliquefaciens* 54 significantly elevated the expression of stress-regulated genes, including late embryogenesis abundant LEA genes, which contribute to cellular protection and molecular stability under water-deficit conditions [112]. In *Brachypodium distachyon* Bd21, inoculation with *B. subtilis* B26 altered the expression of drought-adaptive genes such as *DREB2B*-like, *DHN3*-like, and *LEA-14-A*-like, resulting in enhanced drought tolerance [113]. DREB transcription factors function as central regulators of drought responses by activating downstream protective genes, including LEA and DHN, thereby establishing coordinated stress-response networks. These findings support the concept that microbial colonization preferentially targets regulatory hubs rather than isolated stress genes to improve drought resistance [114]. Comparable regulatory effects were observed in soybean plants inoculated with the drought-tolerant PGPR *P. simiae* strain AU, which enhanced drought tolerance by upregulating genes associated with osmoprotectant biosynthesis (*P5CS* and *GOLS*), water transport (plasma membrane intrinsic proteins and tonoplast intrinsic proteins), and stress-responsive transcriptional regulation [115]. Collectively, these gene expression changes contributed to improved physiological adaptation under drought stress conditions.

In parallel with transcriptional regulation, beneficial microorganisms actively remodel plant phytohormone profiles to favor drought-adaptive physiological states. Key phytohormones, including ABA, ethylene, auxin, cytokinins, gibberellins, and salicylic acid (SA), play critical roles in balancing growth and stress responses during water scarcity [116]. In *Arabidopsis thaliana*, exposure to microbial signaling molecules such as lipo-chitooligosaccharides (LCOs) and thuricin 17 (Th17) induced pronounced hormonal reprogramming under drought stress [114]. LCO treatment reduced levels of IAA, cytokinins, and gibberellins, while increasing ABA and SA concentrations, whereas Th17 similarly elevated ABA and SA levels. These hormonal shifts indicated a microbial-driven transition from growth-promoting pathways toward stress-adaptive signaling dominated by ABA and SA [117]. Furthermore, Curá et al. showed that nitrogen-fixing bacteria *Azospirillum brasilense* SP-7 and *Herbaspirillum seropedicae* Z-152 enhanced growth and stress adaptability in maize [118]. These PGPR strains suppressed the expression of *ZmVP14,* thereby moderating drought-induced elevations in ABA and ethylene levels. Such hormonal modulation mitigated stress-associated hormonal imbalances and improved drought tolerance in maize [119]. Additionally, several beneficial microorganisms alleviate drought stress by degrading the stress hormone ethylene through the activity of ACC deaminase. By lowering ethylene levels and modulating stress-responsive gene expression, ACC deaminase serves as a pivotal microbial trait linking hormonal regulation with downstream drought adaptation mechanisms [120].

### 5.3. Omics Approaches and Epigenetic Regulation of Plant–Microbe Interactions

**Figure 5:**
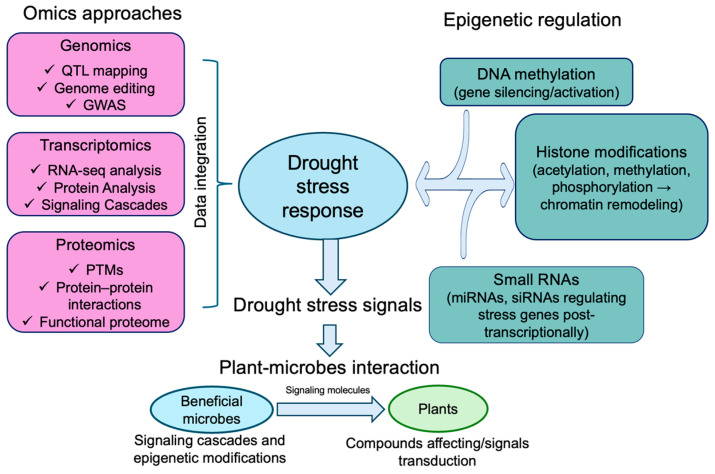
Integrated omics and epigenetic regulation in plant drought stress response and plant-microbe interactions. DNA, deoxyribonucleic acid; GWAS, genome-wide association studies; miRNAs, microRNAs; PTMs, post-translational modifications; QTL, quantitative trait loci; RNA-seq, RNA sequencing; siRNAs, small interfering RNAs. Figure created using Adobe Photoshop (Adobe Systems, San Jose, CA, USA).

Molecular-level analyses of biological systems encompass a wide range of high-throughput approaches, including transcriptomics, proteomics, metabolomics, and genomics. Figure 5 illustrates how multi-omics approaches collectively elucidate plant–microbe interaction networks under drought stress conditions. These technologies provide system-wide insights into genetic, transcriptional, protein, and metabolic profiles, facilitating understanding of plant responses to external stimuli. Integration of multi-omics datasets enables the dissection of complex plant–microbe interactions, particularly under drought stress conditions. Genomic approaches have been applied to identify loci and molecular markers associated with drought resilience. For example, genomic markers have been used to enhance silicon accumulation in rice, contributing to improved drought tolerance [121,122]. Marker-assisted breeding and genome-editing technologies, such as CRISPR/Cas systems, allow functional validation of drought-responsive genomic regions, potentially influencing plant–microbe interaction traits [123,124]. At the microbial level, specific PGPR and rhizobacteria have been reported to harbor drought-related gene clusters involved in osmoprotectant and IAA biosynthesis, which may enhance host drought adaptation under certain conditions [125,126].

It is important to note, however, that these microbial effects are often context-dependent, varying with host species, microbial strain, and environmental conditions. Proteomic analyses have identified stress-responsive proteins and signaling components associated with microbial colonization, such as pathogenesis-related proteins (PR5, PR10), antioxidant enzyme (SOD), and regulatory proteins (CDPKs, PBZ1, OsWRKY30) [127]. Transcriptomic studies reveal dynamic gene expression changes during plant–microbe interactions, including those affecting photosynthesis, carbohydrate metabolism, and stress-responsive transcription factors [128,129,130]. Metabolomics complements these findings by identifying metabolites involved in stress adaptation, which can be modulated by microbial associations [131,132]. Beyond conventional omics, epigenetic regulation has emerged as a promising but still limited mechanism influencing plant–microbe interactions under drought stress. Epigenetic modifications, such as DNA methylation and histone modifications, enable rapid and reversible changes in gene expression without altering DNA sequences, providing phenotypic plasticity in response to microbial signals and environmental cues [133]. A few studies suggested that microbial inoculation can influence plant epigenomes to enhance stress tolerance, for example *Burkholderia phytofirmans* PsJN in potato [134]. and *Bacillus subtilis* B26 in *Brachypodium distachyon* [135]. However, evidence remains scarce, particularly in major crop species, and microbial effects on histone remodeling during drought stress are largely unexplored [136,137,138].

Overall, while multi-omics approaches provide valuable mechanistic insights into plant–microbe interactions, the effects are context-dependent, and the role of epigenetic regulation remains largely speculative. Future studies should combine multi-omics, functional validation, and field trials to establish the reproducibility and scalability of microbial interventions in diverse crops and environmental conditions.

## 6. Future Directions

Drought represents a critical threat to global agricultural productivity and food security, rendering agroecosystems highly vulnerable to ongoing climate change. Harnessing plant–microbe interactions offer a promising strategy to sustain crop productivity under increasing water scarcity. Future research should prioritize the development of robust, field-ready microbial formulations capable of enhancing plant performance under drought conditions while reducing dependence on chemical fertilizers and pesticides. Advancing this field will require integrative approaches that combine signal transduction biology, microbial ecology, and molecular genetics to comprehensively elucidate plant adaptive responses in water-limited environments. Improving the stability, colonization efficiency, and functional consistency of beneficial microbial consortia under field conditions is essential for translating laboratory findings into scalable agricultural applications. Despite recent progress, the complexity of plant-associated microbial communities and their dynamic interactions with host signaling networks remain major challenges to fully understanding microbiome-mediated drought tolerance. Advanced multi-omics platforms, coupled with epigenetic profiling and functional validation of signaling pathways and transcriptional regulators, will be instrumental in uncovering the molecular mechanisms by which microorganisms enhance plant drought resilience. A deeper understanding of these interactions will facilitate the rational design and targeted manipulation of microbial communities to improve crop performance under water stress conditions. Although this research area is still evolving, continued investigation holds significant potential for the development of microbiome-assisted agricultural systems capable of sustaining food production in increasingly arid environments.

## 7. Conclusions

Drought stress remains a major limitation to global agricultural productivity, necessitating innovative, sustainable strategies to enhance crop resilience. This review provides a unique integrative perspective by linking plant morpho-physiological, biochemical, and molecular responses to drought with the regulatory roles of beneficial microbial communities. By synthesizing current knowledge on drought-adaptive microorganisms and their influence on ABA signaling, calcium fluxes, protein kinase cascades, transcriptional regulation, and epigenetic reprogramming, we highlight how plant–microbe interactions can coordinate complex stress responses. Importantly, this work emphasizes the potential applications of these insights for the rational design of microbiome-based bioinoculants and management strategies to promote climate-resilient, resource-efficient agriculture. Future research integrating multi-omics approaches, functional genomics, and rigorous field validation will be critical to translate mechanistic understanding into practical solutions for sustaining crop productivity under water-limited conditions. This review thus bridges conceptual understanding with actionable guidance, offering a roadmap for advancing both fundamental research and applied solutions in plant drought tolerance.

## Figures and Tables

**Table 1:** Representative transgenic plants overexpressing drought-responsive genes and their associated tolerance-enhancing traits.

Donor Plant	Transgenic Plant	Gene(s)	Traits Improved	References
*Pisum sativum*	*Oryza sativa*	*MnSOD*	Increased SOD activity; enhanced oxidative stress protection	[47]
*Arabidopsis thaliana*	*Glycine max*	*AtDREB1A*, *AtDREB2C*, *AtAREB1*	Elevated proline accumulation; enhanced transcriptional activation of drought-responsive pathways	[48]
*Cicer arietinum*	*A. thaliana*	*CarERF116*	Increased LEA gene expression	[49]
*Zea mays*	*Z. mays* mutant	*gl6*	Improved cuticular wax transport; enhanced leaf surface integrity under drought	[50]
*O. sativa*	Transgenic Nipponbare rice (*ERF71* line)	*OsERF71*	Enhanced drought-responsive phenotypic traits	[51]
*Triticum aestivum*	*Nicotiana tabacum*	*TaLEA3*	Regulation of stomatal closure; improved water-use efficiency	[52]
*A. thaliana*	*Brassica napus*	*AtFTA*	Regulation of stomatal closure	[53]
*Mesembryanthemum crystallinum*	*N. tabacum*	*MT1*	Improved photosynthesis	[54]
*Z. mays*	*A. thaliana*	*Chl-NADP-ME*	Enhanced stomatal conductance and biomass under drought stress	[55]
*Hordeum vulgare*	*O. sativa*	*HVA1*	Improved shoot growth, RWC, and water potential	[56]
*Solanum tuberosum*	*Gossypium barbadense*	*StDREB2*	Activation of drought-responsive genes; enhanced antioxidant defense	[57]
*S. lycopersicum*	*S. lycopersicum*	*SlbZIP1*	Increased malondialdehyde regulation; reduced transpiration rate	[58]
*Zingiber officinale*	*N. tabacum*	*ZoCDPK1*	ABA hypersensitivity shown; improved stomatal regulation	[59]
*B. napus*	*B. rapa*	*LEA*	Increased shoot growth and survivability	[60]

ABA, abscisic acid; *AtAREB1*, *Arabidopsis thaliana* ABA-responsive element-binding protein 1 gene; *AtDREB1A*, *A. thaliana* dehydration-responsive element-binding protein 1A gene; *AtDREB2C*, *A. thaliana* dehydration-responsive element-binding protein 2C gene; *AtFTA*, *A. thaliana* farnesyltransferase A gene; *CarERF116*, *Cicer arietinum* ethylene-responsive factor 116 gene; *Chl-NADP-ME*, chloroplastic NADP-dependent malic enzyme gene; *gl6*, glossy6 gene; *HVA1*, late embryogenesis abundant protein 1 gene; *LEA*, late embryogenesis abundant proteins gene; *MnSOD*, manganese superoxide dismutase gene; *MT1*, metallothionein 1 gene; RWC, relative water content; *SlbZIP1*, basic leucine zipper protein 1 gene; SOD, superoxide dismutase; *ZoCDPK1*, calcium-dependent protein kinase 1 gene.

**Table 2:** Role of beneficial microbial species in mitigating drought stress in plants.

Type of Microbes	Microbes	Plants	Beneficial Features	References
Prokaryotic bacteria	*Pseudomonas putida* GAP-P45	*Helianthus annuus*	Produced exopolysaccharides; improved soil aggregation and moisture retention	[67]
	*Bacillus thuringiensis* AZP2	*Triticum aestivum*	Released VOCs that enhanced drought resilience	[68]
	*P. fluorescens*	Various plants	Synthesized IAA; enhanced root growth and nutrient uptake	[69]
	*Paenibacillus polymyxa*	*Arabidopsis thaliana*, Swedish wheat	Produced cytokinins and gibberellins; promoted growth under drought stress	[70]
	*B. licheniformis* strain K11	*Capsicum annuum*	Induced stress-responsive genes and proteins	[71]
	*Burkholderia phytofirmans*; *Enterobacter* sp. FD17	*Zea mays*	Enhanced photosynthesis and biomass accumulation	[72]
	*Rhizobium* sp. strain YAS34	*Phaseolus vulgaris*	Improved nodulation, nitrogen uptake, and ABA accumulation	[73]
	*Pseudomonas* spp.	*Ocimum basilicum*	Enhanced antioxidant defense and osmotic protection	[74]
	*Trichoderma* spp.	*Glycine max*	Secreted enzymes that degraded fungal cell walls; induced plant defense mechanisms	[75]
	*Gluconacetobacter diazotrophicus* Pal5	*Oryza sativa*	Enhanced root architecture and osmotic adjustment	[76]
	*P. aeruginosa* GGRJ21	*Vigna radiata*	Boosted ROS-scavenging enzymes, osmolytes, and RWC; increased root and shoot growth	[77]
	*Shewanella putrefaciens* MCL-1; *Cronobacter dublinensis* MKS-1	*Pennisetum glaucum*	Regulated root development; activated drought-responsive genes	[78]
	*B. subtilis*; *B. thuringiensis*; *B. cereus*	*Glycine max*	Enhanced stomatal conductance and photosynthetic performance	[13]
	*B. subtilis*; *B. thuringiensis*; *B. megaterium*	*Cicer arietinum*	Increased osmolytes, organic acids, and antioxidant enzymes	[79]
	*Bacillus* spp.; *Enterobacter* spp.	*T. aestivum*; *Z. mays*	Enhanced IAA and salicylic acid production	[80]
	*B. subtilis* Rhizo SF48	*Solanum lycopersicum*	Enhanced seed germination and seedling vigor	[81]
	*P. fluorescens* WCS417; *B. amyloliquefaciens* GB03	*Mentha piperita*	Elevated phenolic content and antioxidant protection	[82]
Prokaryotic bacteria + mycorrhizal fungus	*P. chlororaphis* TSAU13 + *Funneliformis mosseae*	*Cucumis sativus*	Increased IAA production; enhanced plant–fungus synergistic protection	[83]
Yeasts + bacteria consortium	*Rhodotorula graminis* + *Burkholderia vietnamiensis*, *R. tropici*, *Acinetobacter calcoaceticus*, *Sphingomonas yanoikuyae*	*Populus deltoids*	Boosted plant development, stress tolerance, and oxidative stress alleviation	[79]
Arbuscular mycorrhizal fungus	*Funneliformis versiformis*	*Z. mays*	Enhanced nutrient absorption, osmolyte accumulation, and antioxidant system	[84]
Bacteria + soil fungus	*Streptomyces laurentii* EU-LWT3–69 + *Penicillium* sp. EU-DSF-10	*Sorghum bicolor*	Enhanced osmolytes, deaminase activity, P-solubilization; reduced lipid peroxidation	[85]
Endophytic fungus	*Neotyphodium coenophialum* (syn. *Epichloë coenophiala*)	*Lolium arundinaceum*; *Nicotiana benthamiana*	Modulated gene expression; improved osmotic balance, antioxidant activity, and leaf gas exchange	[86]

ABA, abscisic acid; IAA, indole-3-acetic acid; ROS, reactive oxygen species; RWC, relative water content; VOCs, volatile organic compounds.

## Data Availability

No new data were created or analyzed in this study. Data sharing is not applicable to this article.
